# Caregiving consequences in cancer family caregivers: a narrative review of qualitative studies

**DOI:** 10.3389/fpubh.2024.1334842

**Published:** 2024-03-22

**Authors:** Masoud Rezaei, Sahar Keyvanloo Shahrestanaki, Razieh Mohammadzadeh, Mohammad Sadegh Aghili, MohammadReza Rajabi, Mohammad Abbasi, Alice Khachian, Reza Momen, Mohammad Khavassi, Simin Aghaei

**Affiliations:** ^1^Nursing and Midwifery Care Research Center, School of Nursing and Midwifery, Iran University of Medical Sciences, Tehran, Iran; ^2^Cardiovascular Nursing Research Center, Rajaie Cardiovascular Medical and Research Center, Tehran, Iran; ^3^Department of Community Health Nursing and Geriatric Nursing, School of Nursing and Midwifery, Tehran University of Medical Sciences, Tehran, Iran; ^4^Faculty of Nursing, Ilam University of Medical Sciences, Ilam, Iran; ^5^Department of Gerontological Nursing, School of Nursing, Abadan University of Medical Sciences, Abadan, Iran; ^6^Department of Cardiology, School of Medicine, Shahed University, Tehran, Iran; ^7^Department of Critical Care Nursing, School of Nursing, Aja University of Medical Sciences, Tehran, Iran; ^8^School of Nursing and Midwifery, Dezful University of Medical Sciences, Dezful, Iran; ^9^Department of Pediatrics, Yasuj University of Medical Sciences, Yasuj, Iran; ^10^Department of Nursing, School of Nursing, Yasuj University of Medical Sciences, Yasuj, Iran; ^11^Student Research Committee, Yasuj University of Medical Sciences, Yasuj, Iran

**Keywords:** Cancer, family caregivers, end of life care, palliative care, caregiving outcome

## Abstract

**Background:**

Cancer is a significant public health issue, causing various challenges for individuals affected by it. As cancer progresses, patients often become dependent on others for support. Family caregivers and members play a crucial role in the recovery and rehabilitation of these patients. However, caregivers themselves face numerous challenges throughout the course of their family member’s illness. Exploring the experiences of family caregivers can inform long-term planning and supportive interventions to address their caregiving difficulties. This study reviews previous literature on caregiving outcomes.

**Method:**

This study presents a narrative review of qualitative studies, analyzing a total of 23 articles. The results were extracted and organized into subcategories. After revision by the research team, main categories were identified. These categories encompass both positive and negative outcomes of caregiving.

**Results:**

The findings of this review demonstrate that caring for a family member with cancer has significant implications for caregivers. These implications include: (A) Positive outcomes of caregiving (such as achieving self-management and balance, promoting kinship intimacy, finding meaning and purpose, and experiencing spiritual growth) and (B) Negative outcomes of caregiving (including care-related physical exhaustion, disruption of personal life plans, psycho-emotional consequences, and socio-economic burden).

**Conclusion:**

The results of this study highlight the challenges faced by family caregivers and emphasize the importance of addressing their needs within the healthcare system. By providing support and attention to their well-being, caregivers can enhance their resilience and adaptability in managing caregiving difficulties.

## Introduction

Cancer is a growing public health concern, with its incidence on the rise (1). It is the second leading cause of death worldwide, accounting for approximately 9.6 million deaths in 2018, or one in six deaths (2). While advancements in cancer treatment have improved survival rates, it has transformed cancer from an acute life-threatening disease to a chronic condition that necessitates long-term care within society (3). In the advanced stages, individuals with cancer experience a decline in performance, an increase in physical and psychological symptoms, and a reliance on others (4). Consequently, family caregivers play a crucial role in supporting these patients and are an integral part of the care process (5). Family caregivers often provide extensive hands-on care for extended periods, without respite, compensation, or external support (6). They fulfill various responsibilities, including physical care, assistance with daily activities, medication management, transportation, emotional support, household chores, and companionship (7, 8). Additionally, family caregivers monitor treatment side effects and symptoms, as well as contribute to care and treatment decisions (9, 10). However, the provision of comprehensive care and support to family members can take a toll on the psychological well-being and quality of life of family caregivers, potentially compromising their health (11–13). Studies have highlighted the lack of support and attention given to family caregivers’ needs (14–16), which may stem from healthcare systems’ limited awareness of the challenges they face. Therefore, gaining a comprehensive and profound understanding of the caregiving experience and subsequent hardships of family caregivers can assist healthcare providers in developing tailored support programs and effective interventions to address these challenges (17). Hence, the objective of this study is to review recent qualitative studies that have examined the issues faced by family caregivers when caring for a terminally ill family member with cancer.

## Methods

In this review, we conducted an electronic search of various databases including PubMed, Science Direct, EMBASE, Web of Science, and Scopus to identify eligible articles published between 2008 and 2023. The search strategy involved using keywords such as “Cancer,” “End of Life Cancer,” “Advanced Cancer,” “Caregiving Consequences,” “Caregiving Impacts,” “Caregiving Experiences,” “Caregiving,” “Caregiver,” “Informal Caregiver,” “Family Caregiver,” “Qualitative Research,” “Qualitative Studies,” and “Qualitative Inquiry.” We also examined the references of retrieved items to find additional relevant articles on the topic.

**Figure 1:**
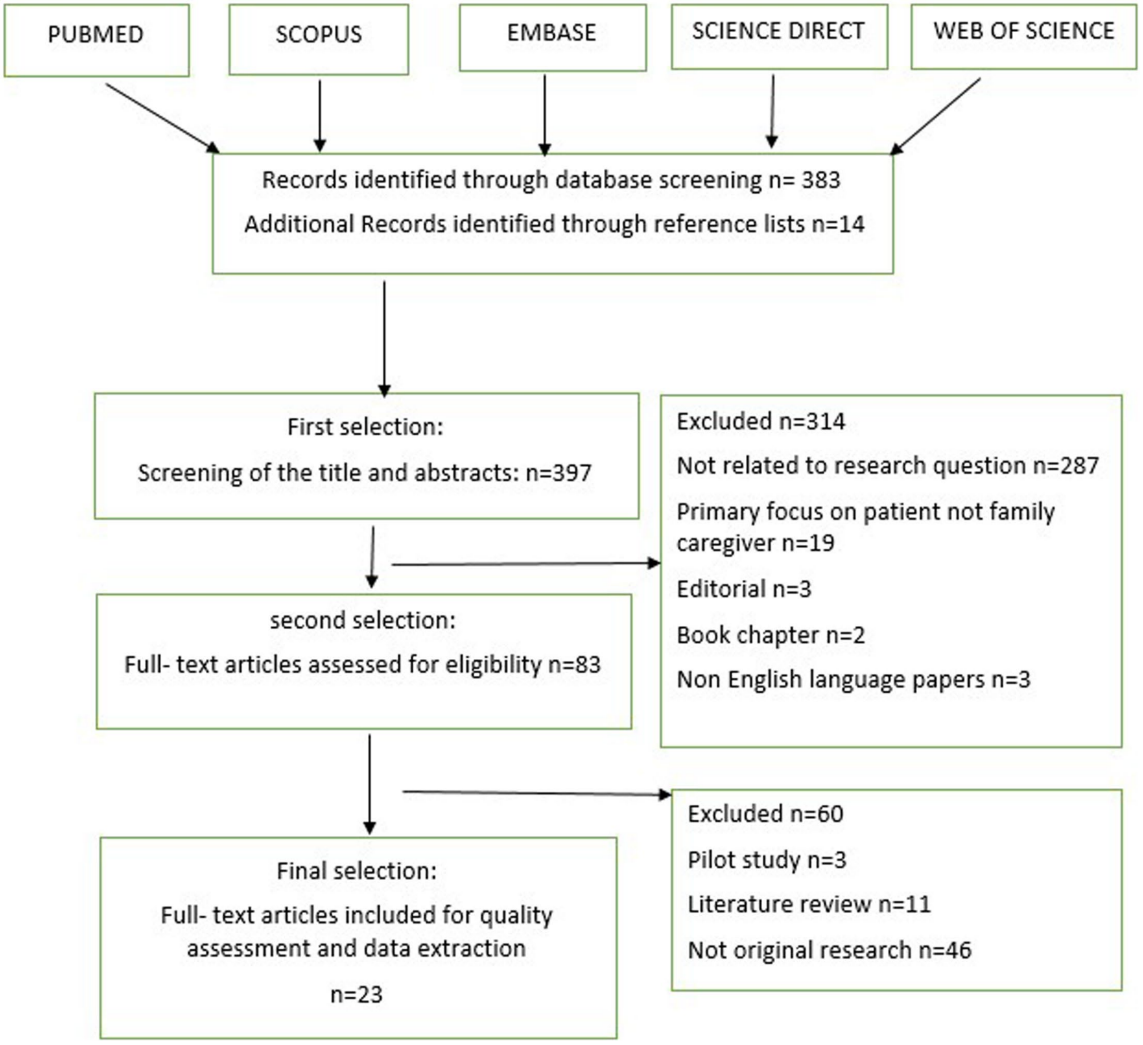
Matrix pattern for qualitative studies selection.

To extract the articles, we employed a matrix pattern of review studies (18, 19) as depicted in Figure 1. Initially, a research group comprising nursing faculty members from Iran University of Medical Sciences was formed. The leader author of this review, (MR), conducted the evidence search. Our aim was to include qualitative studies that employed interview methods, focus groups, or questionnaires to explore the perceptions and experiences of family caregivers regarding the consequences of care. We excluded quantitative or interventional studies, as well as articles that did not utilize primary research methods (e.g., systematic reviews, commentaries, and letters to the editor). Additionally, articles published prior to 2008 were extracted. Documents extracted by other researchers were independently reviewed to ensure the inclusion of relevant and appropriate materials in our research.

Ultimately, we retrieved a total of 397 published articles (395 articles and 2 books) related to the consequences of cancer caregiving on family caregivers. For the final review, we selected 23 qualitative articles. Each retrieved article was thoroughly read by one author and then reviewed by a second author to enhance our understanding of the studies (Table 1).

**Table 1:** Characteristics of included studies.

No	Reference	Country	Study design	Sample Size (FCG)^1^	Age (mean categories)	Cancer type	Main care giving consequence(s)
1	Living in a state of suspension–a phenomenological approach to the spouse’s experience of oral cancer (20)	Sweden	A phenomenological	10	64.5	Oral cancer	lived relation, Negligence of self, A restricted life, Hope in a future, trying to cope with partner’s difficulties, Feelings of sympathy,
2	The Impact of Cancer on Family Relationships Among Chinese Patients (21)	China	Group expressed concerns	84	–	Lung, Breast, Prostate, Stomach, Nasopharyngeal, Pancreatic, Colorectal, Liver, Gynecological, Lymphoma, Leukemia	Suffering, conceal emotion, anxiety, burdening, experience of distress, designing interventions to help patients cope with cancer, calmly, Strengthening, coping
3	What are the perceived needs and challenges of Informal caregivers in home cancer palliative care? Qualitative data to construct a feasible psycho-educational intervention (22)	United Kingdom	Semi-structured qualitative interviews	20		Lung, Prostate, Thyroid, Other (kidney, liver, small intestine)	Uncertainty, Distress, Financial, Challenges with Time, Sleep deprivation, health, Financial Problem, Distress at witnessing disease progression, isolation, Growing resilience and appreciation.
4	Hope against hope: exploring the hopes and challenges of rural female caregivers of persons with advanced cancer (23)	Canada	narrative analysis	23	59	–	Hope, self-care strategies, their emotional journey, well-being, relied on prayer, Hope, hopeful days, Faith, dynamics relationship, self-care strategies to cope
5	Chronicles of informal caregiving in cancer: using ‘The Cancer Family Caregiving Experience’ model as an explanatory Framework (24)	United Kingdom	Semi-structured interviews	53	64.5	Gastrointestinal, Head and Neck, Gynecological, Lung, Breast, Brain, Prostate and Lymphomas	illness-related factors, Care demands, lack of Social support, problem with Employment/finances, Lifestyle change, Appraisal Powerlessness, Hopelessness/helplessness, Entrapment, Future outlook, Health and well-being Mental impact, Physical impact, Cognitive/behavioral Denial, Acceptance, Positive attitude, Maintaining normality, Keep busy, Maintaining normality
6	Oncological patient in palliative care: the perspective of the family caregiver. (1)	Brazil	exploratory and descriptive study with a qualitative approach	12	51	breast cancer ethmoidal breast, larynx, spine, lung, spleen, intestine, cervix, penis, and prostate cancer	Denial, acceptance spirituality Reflecting on the location of the terminal illness: death at home or in the hospital in the perception of the family member Subsidies to support the family member
7	Qualitative analysis of the experience of mental fatigue of family caregivers of patients with cancer in phase I trials (25)	United States	Qualitative analysis	79	–	Advanced cancer	mental fatigue, negative effect on their own self-care, wanted more information and support from professionals
8	The Challenges, Emotions, Coping, and Gains of Family Caregivers Caring for Patients With Advanced Cancer in Singapore (26)	Singapore	qualitative study;	19	46.4	All	caregiving challenges, negative emotions, ways of coping, positive gains of caregiving(Spiritual), Satisfaction, Find meaning of life, Support from family members, increased family closeness, Find a balance, increased knowledge
9	The Spiritual Challenges Faced by Family Caregivers of Patients With Cancer (20)	Iran	qualitative study	21	55.5	All	Spiritual crisis Spiritual distress Disappointment Spiritual coherence Transcendence Appreciation and gratitude Perceiving the protection of God /Seeking God’ satisfaction Being tested by God, trust in God, Appreciation and gratitude, satisfaction
10	Explaining the experiences and consequences of care among family caregivers of patients with cancer in the terminal phase: a qualitative research (27)	Iran	A qualitative content analysis method	18	39	All	Care Challenges and Consequences, Economic Pressures, Physical Problems, Family Challenges, Emotional-Psychological Pressure, Supportive-Palliative Factors, Spirituality, feeling satisfaction from caregiving, increased sense of responsibility towards family, valuing the moments of being with the patient and family, the feeling of getting close to God, getting in touch with death and the transient nature of life, recognizing the value of the parents very existence, spiritual growth and transcendence, and recognizing the love between the patient and self, feeling satisfaction
11	Perceptions of family caregivers of cancer patients about the challenges of caregiving: a qualitative study (14)	Iran	qualitative study	21	44.5	Adenocarcinoma (gastric, uterus, breast, lung, colon and liver), Sarcoma, Leukemia	Confusion Uncertainty disintegration setback new perspective
12	The experiences of family caregivers providing palliative cancer care in Thailand (28)	Thailand	Qualitative study took a phenomenological	14	46.5	All	caring as a team caring as supportive care taking care to keep patients happy, caring for the self while looking after a relative, Trying to be strong, Having unity in the family, Responsibility, Supporting, Balance
13	Caregivers needing care: the unmet needs of the family caregivers of end-of-life cancer patients (29)	Iran	semi-structured interviews, content analysis	18		All	social needs (support for care, effective communication and financial support.), cognitive needs (comprised of educational support and support in decision-making), and psychological needs (support for psychological trauma, preparation to confront the reality of the death of a loved one, and support for mourning). Preparation to confront. Effective communication
14	Family Caregivers’ Perspectives on Communication with Cancer Care Providers (30)	Colombia	thematic analysis of qualitative	63	–	All cancer types	sensitive to unmet information needs, and responsive to the potentially different communication preferences of patients and caregivers. Responsive
15	A Qualitative Study on Cancer Care Burden (31)	Iran	Qualitative study	16	39.3	Different types of cancer	-burnout (physical problems and psych emotional stress), − role conflict (balancing caring roles and family responsibilities; failure in professional or educational roles), − health system tensions (inadequate support from health professionals; ignorance of family members in health structure), − social challenges of cancer economic burden; taboo of cancer. - Balancing caring roles and family responsibilities
16	The experience of family caregivers of patients with cancer in an Asian country: A grounded theory approach (5)	Indonesia	Grounded theory	24	–	Breast, Ovarian, Cervix, Others	Physical impact/psychological impact/ financial impact/ Social impact/ Sacrifices/ Coping
17	Cancer family caregivers’ quality of life and the meaning of leisure (32)	South Korea	A Qualitative Study	10	-	-	stress process, Caregivers invest a high level of effort in adapting to their situations, Positivity compassionate care, Memory of meaningful times, LACK OF Leisure, Leisure Experiences, Adapting
18	The role, impact, and support of informal caregivers in the delivery of palliative care for patients with advanced cancer: A multi-country qualitative study (33)	Sub-Saharan Africa	A multi-country qualitative study	48	47	–	caregivers are coordinators of emotional, practical, and health service matters, caregiving comes at a personal social and financial cost, practical and emotional support received and required, experience of interacting and liaising with palliative care services, medical, physical, financial, and emotional needs, interacting and liaising, improving
19	Adapting ENABLE for patients with advanced cancer and their family caregivers in Singapore: a qualitative formative evaluation (34)	Singapore	Qualitative formative evaluation with a thematic analysis approach	31	–	Advanced Cancer	Financial Problem, Frequency and duration of sessions to be kept flexible, sexuality is an issue, experience psycho-emotional struggles,
20	Challenges of Help-Seeking in Iranian Family Caregivers of Patients with Cancer: A Qualitative Study (35)	Iran	Qualitative study	15	37.93	–	being strained by social desirability; stigmatizing attitudes toward help-seeking reactive self-forgetfulness resistance to change
21	Assessing the Comprehensive Training Needs of Informal Caregivers of Cancer Patients: A Qualitative Study (36)	Canada	Phenomenological Design	7	53.1	Head and Neck, Breast and Thyroid	significant emotional strain, more practical information, expressed the desire for greater social support, lack of Social Supports, Preparedness and Duties
22	Family caregivers’ lived experience of caring for hospitalised patients with cancer during the COVID-19 lockdown: A descriptive phenomenological study (13)		A Descriptive phenomenological Approach	20	–		Feeling scared for the patient, Living a life feeling trapped, Feeling neglected and unseen, Growing resilience and appreciation.
23	The long-term experience of being a family caregiver of patients surgically treated for esophageal cancer (37)	Sweden	A Qualitative Descriptive Study	13	–	Esophageal Cancer	The most essential/evident stress factors for the family caregivers were dis-tress regarding the patients’ nutrition, fear of tumor recurrence and worry about the future. a transition was experienced, going from a family member to a caregiver, and the many psychosocial aspects of this transition. Rewards and benefit, prioritization of life, Health and wellbeing, Self-concept, Changing roles and relationship, Coping

^1^Family Caregiver.

## Results

**Figure 2:**
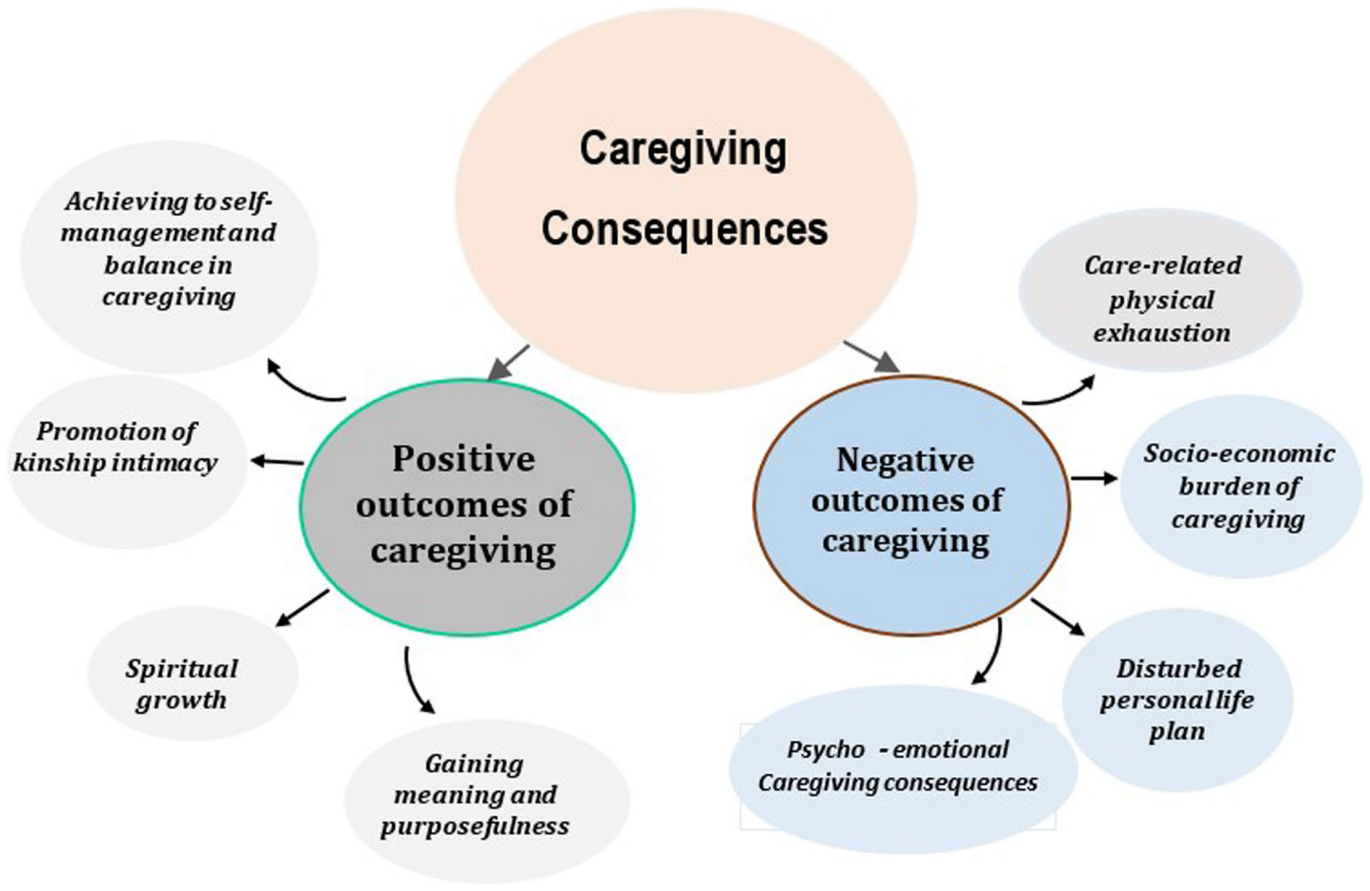
Caregiving consequences in cancer family caregivers.

Twenty-three qualitative studies were included in the review, encompassing various designs such as phenomenological, thematic analyses, content analysis, grounded theory, qualitative descriptive study, and narrative analysis. These studies were conducted in different countries, including the United States, United Kingdom, Canada, Sweden, Colombia, Brazil, Sub-Saharan Africa, Iran, Singapore, Vietnam, South Korea, Indonesia, China, and Thailand. The data was collected through semi-structured interviews and focus group discussions, involving a total of 639 participants. The findings of these studies shed light on the impact of caring for family caregivers of cancer patients. The review process involved extracting data related to the research question and organizing it into sub-categories. These sub-categories were then reviewed by the research team, leading to the identification of new main categories that describe the positive and negative outcomes of caregiving. The positive outcomes include achieving self-management and balance, promoting kinship intimacy, gaining meaning and purposefulness, and experiencing spiritual growth. On the other hand, the negative outcomes encompass care-related physical exhaustion, disturbed personal life plan, psycho-emotional consequences of caregiving, and socio-economic burden (Figure 2).

## Positive outcomes of caregiving

### Achieving to self-management and balance

The experiences of family caregivers in studies showed that one of the important aspects of caregiving for their family member is achieving self-management and balance in the care trajectory, which was extracted from 13 sub-categories (Table 2).

**Table 2:** Emerged categories and sub-categories.

Related articles	Sub- categories	Main categories	
(14, 20, 22, 27, 31)	physical tiredness, Sleep deprivation, Caregiver health challenges, Physical Problems, Burn out, Physical Problems, Exhaustion	Care-related physical exhaustion	Positive outcomes of caregiving
(13, 20, 21, 23, 28–33, 37)	Trying to cope with partner’s difficulties, Strengthening support system for coping, Self-care strategies to cope, Maintaining normality, Find a balance, Increased knowledge, Responsibility, Supporting, Balancing caring roles and family responsibilities, Adapting, Coping, Growing resilience and appreciation, Trying to be strong, responsive to the potentially different communication	Achieving to self-management and balance in caregiving	
(26, 28)	Support from family members, increased family closeness, Having unity in the family	Promotion of kinship intimacy	
(26, 27)	Satisfaction, Find meaning of life, feeling satisfaction	Gaining meaning and purposefulness	
(1, 20, 23, 26)	Faith, Acceptance, spirituality, Spiritual support, faith and trust in God, appreciation and gratitude and seeking God’s satisfaction	Spiritual growth	
(14, 20, 35)	Negligence of self, changed plane, restricted life space, additional responsibilities at home, changed living routines, Standby, Overestimating Caring Responsibilities	Disturbed personal life plan	Negative outcomes of caregiving
(5, 14, 20, 21, 25, 27, 29, 31, 36)	Worry, psychological distress, emotional turmoil, emotional challenges, feeling undermined and unappreciated, inability to justify, punishment, Emotional Inhibition, Emotional-Psychological Pressure, Feeling inadequate, Feeling instability, Hopelessness, Caregiving tension, psychological trauma,Psycho-emotional stress, feeling trapped, mental fatigue, significant emotional strain	Psycho-emotional consequences of caregiving	
(24, 27, 31, 34, 35)	Role conflict, economic burden, Relationships, Social Support, Employment, Inadequate support, Ignorance, Social challenges, Economic burden, Failure in professional or educational roles, Economic Pressures, social desirability, Stigmatizing attitudes, Financial Problem	Socio-economical burden of caregiving	

In the study by Stamataki et al. (24) adopting a positive attitude and maintaining normalcy are coping mechanisms that help caregivers manage the stress of providing care to a family member with cancer. Caregivers often emphasized the importance of maintaining a positive attitude or fighting spirit, regardless of how they were actually feeling, especially after diagnosis. In this study, the need to be strong in front of the family member was mentioned, which helped the caregivers to manage their emotions (24). Another thing that seems to make it possible for family caregivers to self-manage and achieve balance during caregiving is maintaining hope in the caregiving process, which is mentioned in study by William’s et al. (23). The participants in the mentioned study stated that while caring for a family member, they hope for a better tomorrow will come in which their patient will not be in pain, sleep comfortably, and the patient’s body will be strong against heavy treatments (23). Also, it was reported in another study that a caregiver bonds with the patient and not only feels sympathy, but empathy and understanding. Developing a consistent and compassionate approach to caregiving is important. Because failure to adapt can lead to compassion fatigue, in which the caregiver is unable to cope with stress and avoid physical, psychological, spiritual, or social exhaustion. In addition, adapting to the conditions can create a calm and stress-free environment for a cancer patient (32). In another study, it is stated that family caregivers learned to let go of their stress and frustration in order to adapt to negative emotions over time. They gradually find a balance between caregiving and other commitments so that other family members are not neglected or overwhelmed by caregiving (26).

### Promotion of kinship intimacy

Another important outcomes of caregiving of a family member with cancer was promotion of kinship intimacy of other family members, which was extracted from 3 sub-categories (Table 2). In a study by Wannapornsiri (28) caring as a team involved integrating family members in to a care team, sharing caregiving responsibilities, and creating family unity. The disease process and cancer treatment take a long time, so family caregivers come together to care for and take responsibility for their loved ones. Care was provided by a team of family members and family caregivers have to deal with the unpredictability of their loved one’s illness as well as the related costs, and there was a sense of family unity among the members regarding these issues (28).

In the study by Leow and Chan (26) mentioned the family caregivers described feeling more close to the family member with advanced cancer because they spent more time with the patient as a result of the caregiving. Some caregivers also reported that they have established a closer relationship with other family members since a family member has been diagnosed with cancer (26).

### Gaining meaning and purposefulness

Another important positive outcomes of caregiving for family caregiver was gaining a sense of meaning and purposefulness in cancer care journey. In the study by Leow and Chan (26) it is well noted that through caregiving, family caregivers realized their importance in caring for their loved one as well as their own lives. They felt satisfied that other family members were healthy and their family relationships. They were more determined to live life to the best of their abilities. Caregivers realized that happiness in life comes from being with and caring for their loved ones. Caregiving also changed their perception of life. They realized that all humans, regardless of how strong, famous, or successful they had been in the past, would go through the same process of illness and weakness (26). In addition, the study of Arian et al. (27) reported that taking care of a family member with cancer increases the sense of responsibility towards the loved one, valuing the moments of being with the patient and the family, feeling self-satisfaction and a sense of value with care, which can show It should give a sense of meaning and purpose in the path of care (27).

### Spiritual growth

In past studies, it was found that the experience of caring for a family member strengthened the spirituality and strengthened the caregivers’ relationship with God. The main category of spiritual growth was extracted from 6 sub- categories, which are given in the Table 2.
In the study by Williams et al. (23), some participants found hope with faith and belief in God and that their fate is in the hands of God or something greater than themselves. They relied on prayer to get through the day and overcome difficulties and attributed their ability to cope and get through each day to their faith (23). In addition, in the study by Albuquerque et al. (1), it was found that family caregivers seek refuge in God to get rid of the suffering and emotional pain caused by the suffering of their own patients. So that faith in God appears as one of the pillars of strength, security and support for families who cope with the situation and helps to accept the final process (1). In another study, it was stated that the experience of caring for a family member and the ups and downs of caring have led to the creation of a spiritual coherence, achieving inner peace, faith and trust in God, and uniformity in a person’s belief system (14).

## Negative outcomes of caregiving

### Care related physical exhaustion

One of the most important and common problems of family caregivers when caring for a family member with cancer was physical fatigue caused by caregiving, which was extracted from the 7 sub-category of other studies (Table 2).

The results of Stamataki et al.’s study (24) showed that caregivers were damaged in terms of health and physical well-being. Also, in another study, it was stated that health care workers should also deal with their physical health in addition to sleep problems. They reported health problems such as back pain, poor mobility, stomach ulcers and illness (22). In the study of Hassankhani et al. (31) reported that many families reported a variety of health problems resulting from their role as long-term caregivers. These physical problems mentioned included pain in specific areas such as knees, wrists and stomach. General body pain; sleep disorders; weight loss and appetite; reduction of physical difficulties; and the weakness of the immune system was one of the other physical problems that the caregivers experienced during the family member’s illness.

### Disturbed personal life plan

In past studies, it was found that the families of cancer patients who are directly involved with the family member’s illness, their routine life plan is disturbed and all their life plans must be adjusted with the problems of the family member’s disease and treatment. This main category can be described with 7 sub-categories from past studies (Table 2).

In Nemati et al. (37) study, family caregivers have stated that they have always been in a state of alertness and have been constantly waiting to change their life routines according to the physical and mental condition of the family member. This change in the trends and plans of life has brought an unpleasant state of uncertainty for them. In another study, it was stated that the overestimation of caregiving obligations has led to an overemphasis on caregiving activities. By carrying out intensive care activities, the patient becomes too dependent on the caregiver, and the caregivers are unable to meet their needs due to the priority of the family member’s needs. So that they are so immersed in the role of caregiver that they forget their life plans (35). In another study, family caregivers reported that cancer diagnosis and treatment represent sudden changes in their family member’s life. Because they put their lives on hold and adapt their lifestyles to the physical needs and limitations of their patients. The start of treatment may stop their normal life, as plans for vacations, spending time with their family member in the near future will have to change (20).

### Psycho-emotional consequences of caregiving

The psychological and emotional consequences of caregiving seem to be the most important problem facing a family with a cancer member. Because it can affect other aspects of life. This main category was obtained from the 18 sub-categories found in other studies, which is given in Table 2.

In a study, it was stated that during the treatment of a patient with oral cancer, the patient’s spouse in the role of caregiver faces the possibility of losing his life partner. As a result, caregivers may suppress thoughts about their own physical and mental health due to emotional reactions to their partner’s illness and treatment. Caregiver’s emotional concerns may appear at the very beginning of sampling time or waiting to receive the news of their partner’s cancer diagnosis (20). In another study that asked the opinions of patients about the problems of their caregivers. They stated that although they experienced psychological distress associated with their cancer diagnosis, they found that such emotional turmoil was also common among their family caregivers. So that family caregivers, when they have witnessed the pain and suffering of their loved ones, suffer at the same time as their patients, which is called equal suffering or family distress in the mentioned study (21). In William et al. (23) study, it was reported that participants spoke significantly about the emotional aspects of the caregiving experience, and it was clear that they experienced a range of emotions. The participants’ emotional experience included fear, worry, sadness, guilt, helplessness, anger, loneliness, empathy, love, and gratitude. It was also found that the participants were generally afraid of the future and uncertainty about the status of their loved ones and their lives. They expressed concern about specific things, such as how the care recipient would respond to treatment, and worry and guilt about any time they were away from the care recipient. They expressed their sadness about losing their former life and their loved one’s life and imagining life without that person. These fears and worries could destroy hope (23). In the study of Arin et al., they express the origin of emotional and psychological problems of caregivers in a different way. These problems can be related to the physical problems of the patient; It can be related to concerns about the future after the death of the patient for other family members, or it can be related to concerns about the caregivers themselves, who may possibly lose a strong source of support in the future. In addition, depression and discomfort, irritability, reduced threshold of tolerance, temporary forgetfulness, numb feeling, pitiful look of relatives, feeling alone and caregiving pressure have been bothersome for them when caring for a family member (27).

### Socio-economical burden of caregiving

Socio-economic problems are other consequences of caring for a family member with cancer, which have been raised by numerous studies of the experiences of family caregivers Table 2.

In the study by Hassankhani et al. (31), it highlights the impact of cancer on financial and social consequences and shows that families with a family member suffering from cancer especially in eastern societies, it is men who are responsible for earning and supporting the family and if men get cancer, it affects the economic problems of the whole family. It was also mentioned that family caregivers enter the caregiving role without any preparation and becoming a family caregiver leads to an increase in the workload and as a result decreases the effectiveness in occupational and educational responsibilities and affects the social roles of family caregivers. As a result, balancing caregiving roles and family responsibilities in the early stages of illness was an important concern mentioned by participants who are family caregivers. Caregivers are sometimes confused in roles and responsibilities and cannot balance caregiving duties with other daily responsibilities (31).

Stamataki et al. (24) in their study mentioned relationships, social support, employment and lifestyle as secondary stressors. And these are defined as stressors that do not originate from caregiving, but are influenced by role demands. The experiences of caregivers in the mentioned study show that maintaining friendships, social relationships and social life has been challenging for most of them. So that at the beginning of the diagnosis, some caregivers decided to isolate themselves from their social network to avoid discussing the disease with others. In addition, some participants in the aforementioned study reported that their daily schedule and lifestyle were affected as a result of their caregiving role, and that during the active treatment phase of the family member, they did not want to plan and their life was put on hold (24).

In another study, family caregivers have stated that they have experienced a financial and job crisis. Financial pressures include running out of savings, financial and insurance problems, transporting patients to medical centers. Job problems have also been significant, so that self-employed caregivers have left their jobs and government-employed caregivers have faced certain job challenges (27).

In addition to the economic problems that arise for the family, in some cultures and social structures, it is difficult for family members to ask for help from others. So that caregivers are forced to act according to social standards and norms in order not to be judged by the society and to feel safe in their social life. This view leads to the isolation of family caregivers because they prefer to rely on themselves instead of asking others for help with problems (38).

## Discussion

In this study, we investigated the consequences of caring for a family member with cancer in family caregivers in different communities. The results of the review of these studies showed that in the process of caregiving for a patient with cancer, caregivers gain experiences that have different effects on their lives. These experiences were categorized into positive and negative outcomes, where positive outcomes of care included Achieving to self-management and balance in caregiving, Promotion of kinship intimacy, gaining meaning and purposefulness, Spiritual growth and negative outcomes of care included Care-related physical exhaustion, Disturbed personal life plan, Psycho-emotional consequences of caregiving, Socio-economic burden of caregiving.

By inferring from the results of the selected studies, it was found that families with a person with cancer try to achieve balance and self-management as soon as possible with different approaches and the facilities they have in order to adapt to the problems of care and illness. Because they know that the first and most important sources that can help the patient to solve the problems of the disease are family members who have taken direct and full-time care. They try to maintain a positive attitude and maintain hope, apply self-care techniques, practice resilience and stay strong in the path of care (13, 14, 23–26, 28, 32). Another consequence of caring for a loved one with cancer for families seems to be the promotion of intimacy, unity and solidarity between family members and other relatives. So that the review of studies showed that the problem that arose for one family member brought other family members closer together and the problems that arose for the patient led to family distress. And the family has experienced the pain and suffering along with the family member with cancer. Therefore, all family members and relatives, by helping each other and dividing the tasks, try to make the care burden easier for each other and reduce this distress in the family (26, 28). In the review of the studies, it was found that the family caregiver with the experience of the role of caregiver and assuming the responsibility of handling the illness and treatment of the family member has a sense of self-satisfaction and gaining a sense of meaning and purpose for them. Also, they have understood that they can be useful for the family. This sense of self-satisfaction helped them maintain hope and stay on the path of caregiving (26, 27). Understanding the existence of God in all periods of the illness of a family member with cancer was one of the other things that can be mentioned as a positive outcome in the studies. So that the occurrence of such problems on the way of the families strengthened the faith and belief in the existence of a greater power within the caregivers so that they sought refuge in God to achieve inner peace and freedom from problems. Spiritual coherence is perhaps the best word to describe spiritual growth as a positive outcome of caregiving in family caregivers (1, 14, 23, 26, 37). In relation to the negative consequences of caregiving, the results of some studies showed that family caregivers ignore their basic needs in order to help the family member and forget themselves in this difficult and uneven path, and this neglect of their needs leads to fatigue. It will become physical and exhausting, and caregivers face many challenges related to their physical health, such as lack of sleep, anorexia, and back pain, which can affect their quality of life (14, 20, 22, 27, 31). Disruption of personal life routines and adjustment of the caregiver’s life time with the family member’s illness and suffering were other issues highlighted by the results of the studies. In such a way that prioritizing the needs of the family member has caused the caregivers to stay away from other life plans and spend all their time on providing medicine, going to and from medical facilities and taking care of household affairs. Therefore, being confined in the triangle of home, pharmacy and hospital and not taking care of personal life plans were other experiences of caregivers in the caregiving trajectory (14, 20, 35). Emotional and psychological pressures there were other cases that previous studies have mentioned and the results of these studies show that the most important negative consequence of caregiving for a cancer patient can be emotional and psychological problems, and family members have many fears and worries during this period. They have experienced fear of worsening of the disease and ineffectiveness of the treatment and fear of treatment complications. Furthermore, worry and anticipation of the death of a family member and the problems that the family may experience after the death of a family member can indicate the occurrence of mental and emotional turmoil in the caregivers (5, 14, 20, 21, 27, 31, 37, 38). The social and economic consequences of cancer are perhaps the biggest problem for families with a person with cancer. Because it can affect other aspects of care. So that in the studies it was found that in addition to incurring heavy costs for the treatment of the disease, family members have to leave their jobs for full-time care and focus entirely on the illness of the family member. But the consequences of leaving a job can lead to a decrease in income, withdrawal from social roles, or social isolation (24, 27, 30, 31, 35).

## Conclusion

The results of this study showed that family caregivers of people with cancer had different experiences of caring for a family member. Trying to manage problems and achieve balance in the caregiver, gaining intimacy with other family members and relatives, gaining a sense of meaning and purpose and spiritual growth, physical fatigue related to caregiving, failure in personal life plan, psychosocial consequences of caregiving and socioeconomic consequences of caregiving was the most important outcomes in the literature. According to the obtained results, it is important to pay attention to several points. 1- Family caregivers suffer along with their family members and should be considered as second-ordered patients. In this way, paying attention to their physical and psychological needs should be the priority of holistic care in the health care system. 2- Family caregivers in different cultures may have a different understanding of caregiving experiences, so in some cultures, cancer disease is considered a social stigma, and therefore it is not easy for them to seek help and accept help in caregiving. Therefore, they bear the problems alone.so it is necessary to consider measures in the health care system, especially in palliative care environments. So that the families of caregivers can narrate their problems without fear and worry and accept the support of others easily. 3- Given that family caregivers with the lowest level of information and support, they embark on the unknown and ups and downs path of caregiving. It seems that there is a need to provide conditions for these caregivers to receive guidance from their peers who have had the experience of caring for their loved one. Do in such a way that associations for health vows or sympathy vows consisting of family caregivers are formed.

## Author contributions

MRe: Conceptualization, Writing – original draft, Writing – review & editing. SK: Data curation, Formal analysis, Investigation, Methodology, Validation, Writing – original draft. RaM: Investigation, Writing – original draft, Writing – review & editing. MAg: Investigation, Methodology, Validation, Writing – original draft. MRa: Formal analysis, Methodology, Supervision, Writing – original draft. MAb: Resources, Supervision, Visualization, Data curation, Writing – original draft. AK: Conceptualization, Data curation, Project administration, Resources, Validation, Visualization, Writing – original draft. ReM: Conceptualization, Resources, Supervision, Validation, Writing – original draft. MK: Methodology, Project administration, Resources, Validation, Writing – original draft. SiA: Formal analysis, Investigation, Methodology, Writing – original draft.

## References

[1] de Albuquerque Pinheiro ML Pimpão Martins FD de Oliveira Rafael CM Tupinambá Silva De Lima U. Oncological patient in palliative care: the perspective of the family caregiver. J Nurs. (2016) 10:1749. doi: 10.5205/reuol.9003-78704-1-SM.1005201622

[2] Cancer IAfRo. The global Cancer observatory GLOBOCAN database 2020. Geneva: World Health Organization (2020).

[3] Ganz PA Rowland JH Desmond K Meyerowitz BE Wyatt GE. Life after breast cancer: understanding women's health-related quality of life and sexual functioning. J Clin Oncol Off J Am Soc Clin Oncol. (1998) 16:501–14. doi: 10.1200/JCO.1998.16.2.501, PMID: 9469334

[4] Rolland JS. Cancer and the family: an integrative model. Cancer. (2005) 104:2584–95. doi: 10.1002/cncr.21489 16270342

[5] Kristanti MS Effendy C Utarini A Vernooij-Dassen M Engels Y. The experience of family caregivers of patients with cancer in an Asian country: a grounded theory approach. Palliat Med. (2019) 33:676–84. doi: 10.1177/0269216319833260, PMID: 30916614 PMC6537031

[6] Lynn J. Strategies to ease the burden of family caregivers. JAMA. (2014) 311:1021–2. doi: 10.1001/jama.2014.1769 24618963

[7] Glajchen M. 33 role of family caregivers in cancer pain management. Cambridge: Cambridge University Press (2009).

[8] Given BA Given CW Sherwood P. The challenge of quality cancer care for family caregivers. Semin Oncol Nurs. (2012) 28:205–12. doi: 10.1016/j.soncn.2012.09.002 23107177

[9] Van Ryn M Sanders S Kahn K Van Houtven C Griffin JM Martin M. Objective burden, resources, and other stressors among informal cancer caregivers: a hidden quality issue? Psycho-Oncology. (2011) 20:44–52. doi: 10.1002/pon.1703, PMID: 20201115 PMC4479404

[10] Gray TF Nolan MT Clayman ML Wenzel JA. The decision partner in healthcare decision-making: a concept analysis. Int J Nurs Stud. (2019) 92:79–89. doi: 10.1016/j.ijnurstu.2019.01.006, PMID: 30743199

[11] Carretero S Garcés J Ródenas F Sanjosé V. The informal caregiver's burden of dependent people: theory and empirical review. Arch Gerontol Geriatr. (2009) 49:74–9. doi: 10.1016/j.archger.2008.05.004, PMID: 18597866

[12] Schulz R Sherwood PR. Physical and mental health effects of family caregiving. J Soc Work Educ. (2008) 44:105–13. doi: 10.5175/JSWE.2008.773247702 PMC2791523 18797217

[13] Ma H Zhao T Ma Y Yuen JWM Ka Yan H Yung JY. Family caregivers' lived experience of caring for hospitalised patients with cancer during the COVID-19 lockdown: a descriptive phenomenological study. J Clin Nurs. (2023) 32:7509–18. doi: 10.1111/jocn.16817, PMID: 37370254

[14] Nemati S Rassouli M Ilkhani M Baghestani AR. Perceptions of family caregivers of cancer patients about the challenges of caregiving: a qualitative study. Scand J Caring Sci. (2018) 32:309–16. doi: 10.1111/scs.12463 28869659

[15] Paavilainen E Salminen-Tuomaala M Kurikka S Paussu P. Experiences of counselling in the emergency department during the waiting period: importance of family participation. J Clin Nurs. (2009) 18:2217–24. doi: 10.1111/j.1365-2702.2008.02574.x, PMID: 19583653

[16] Omari FH. Perceived and unmet needs of adult Jordanian family members of patients in ICUs. J Nurs Scholarsh. (2009) 41:28–34. doi: 10.1111/j.1547-5069.2009.01248.x, PMID: 19335675

[17] Lund L Ross L Petersen MA Groenvold M. Cancer caregiving tasks and consequences and their associations with caregiver status and the caregiver’s relationship to the patient: a survey. BMC Cancer. (2014) 14:1–13. doi: 10.1186/1471-2407-14-541 25069703 PMC4122762

[18] Coughlan M Cronin P. Doing a literature review in nursing, health and social care. London: Sage (2016).

[19] Akbari M Alavi M Irajpour A Maghsoudi J. Challenges of family caregivers of patients with mental disorders in Iran: a narrative review. Iran J Nurs Midwifery Res. (2018) 23:329–37. doi: 10.4103/ijnmr.IJNMR_122_17, PMID: 30186336 PMC6111657

[20] Röing M Hirsch JM Holmström I. Living in a state of suspension–a phenomenological approach to the spouse’s experience of oral cancer. Scand J Caring Sci. (2008) 22:40–7. doi: 10.1111/j.1471-6712.2007.00525.x, PMID: 18269421

[21] Lee J Bell K. The impact of cancer on family relationships among Chinese patients. J Transcult Nurs. (2011) 22:225–34. doi: 10.1177/1043659611405531 21536787

[22] Harding R Epiphaniou E Hamilton D Bridger S Robinson V George R. What are the perceived needs and challenges of informal caregivers in home cancer palliative care? Qualitative data to construct a feasible psycho-educational intervention. Support Care Cancer. (2012) 20:1975–82. doi: 10.1007/s00520-011-1300-z, PMID: 22072049

[23] Williams A Duggleby W Eby J Cooper RD Hallstrom LK Holtslander L. Hope against hope: exploring the hopes and challenges of rural female caregivers of persons with advanced cancer. BMC Palliat Care. (2013) 12:1–10. doi: 10.1186/1472-684X-12-44 24341372 PMC3878500

[24] Stamataki Z Ellis J Costello J Fielding J Burns M Molassiotis A. Chronicles of informal caregiving in cancer: using ‘the Cancer family caregiving Experience’model as an explanatory framework. Support Care Cancer. (2014) 22:435–44. doi: 10.1007/s00520-013-1994-1, PMID: 24091719

[25] Washington KT Craig KW Oliver DP Ruggeri JS Brunk SR Goldstein AK. (2019). Family caregivers’ perspectives on communication with cancer care providers. J Psychosoc Oncol. 37:777–790. doi: 10.1080/07347332.2019.1624674 31204604 PMC7350905

[26] Leow MQ Chan SW. The challenges, emotions, coping, and gains of family caregivers caring for patients with advanced cancer in Singapore: a qualitative study. Cancer Nurs. (2017) 40:22–30. doi: 10.1097/NCC.0000000000000354, PMID: 26925989

[27] Arian M Younesi SJ Khanjani MS. Explaining the experiences and consequences of care among family caregivers of patients with cancer in the terminal phase: a qualitative research. Int J Cancer Manag. (2017) 10:e10753. doi: 10.5812/ijcm.10753

[28] Wannapornsiri C. The experiences of family caregivers providing palliative cancer care in Thailand. Int J Palliat Nurs. (2018) 24:559–65. doi: 10.12968/ijpn.2018.24.11.559, PMID: 30457461

[29] Weiss DM Northouse L Duffy S Ingersoll-Dayton B Katapodi M LoRusso PM. (2016). Qualitative analysis of the experience of mental fatigue of family caregivers of patients with cancer in phase I trials. Oncol. Nurs. Forum. 43:E153–E160. doi: 10.1188/16.ONF.E153-E160 27314198

[30] Yang GM Dionne-Odom JN Foo YH Chung AHM Kamal NHA Tan L. Adapting ENABLE for patients with advanced cancer and their family caregivers in Singapore: a qualitative formative evaluation. BMC Palliat Care. (2021) 20:1–11. doi: 10.1186/s12904-021-00799-y 34158022 PMC8218975

[31] Hassankhani H Eghtedar S Rahmani A Ebrahimi H Whitehead B. A qualitative study on cancer care burden: experiences of Iranian family caregivers. Holist Nurs Pract. (2019) 33:17–26. doi: 10.1097/HNP.0000000000000309 30422921

[32] Lim J Cho H Bunds KS Lee C-W. Cancer family caregivers’ quality of life and the meaning of leisure. Health Care Women Int. (2021) 42:1144–64. doi: 10.1080/07399332.2020.1752214, PMID: 32490741

[33] Adejoh SO Boele F Akeju D Dandadzi A Nabirye E Namisango E. (2021). The role, impact, and support of informal caregivers in the delivery of palliative care for patients with advanced cancer: A multi-country qualitative study. Palliat Med. 35:552–562. doi: 10.1177/0269216320974925 33353484 PMC7975852

[34] Ringborg CH Wengström Y Schandl A Lagergren P. (2023). The long‐term experience of being a family caregiver of patients surgically treated for oesophageal cancer. J Adv Nurs. 79:2259–2268. doi: 10.1111/jan.15580 36779443

[35] Hamedani B Alavi M Taleghani F Fereidoonimoghadam M. Challenges of help-seeking in Iranian family caregivers of patients with Cancer: a qualitative study. Int J Cancer Manag. (2022) 15:e127060. doi: 10.5812/ijcm-127060

[36] Papadakos J Ugas M Quartey NK Papadakos C Giuliani ME. (2023). Assessing the comprehensive training needs of informal caregivers of cancer patients: a qualitative study. Curr. Oncol. 30:3845–3858. doi: 10.3390/curroncol30040291 37185404 PMC10137188

[37] Nemati S Rassouli M Baghestani AR. The spiritual challenges faced by family caregivers of patients with cancer: a qualitative study. Holist Nurs Pract. (2017) 31:110–7. doi: 10.1097/HNP.0000000000000198, PMID: 28181976

[38] Hashemi M Irajpour A Taleghani F. Caregivers needing care: the unmet needs of the family caregivers of end-of-life cancer patients. Support Care Cancer. (2018) 26:759–66. doi: 10.1007/s00520-017-3886-2 28952034

